# 659. Geographical Clustering of the Likelihood of Treatment in Histologically Diagnosed *Helicobacter pylori* in the BOSTON-HP Cohort

**DOI:** 10.1093/ofid/ofad500.722

**Published:** 2023-11-27

**Authors:** Marco Noriega, Christina Lee, Joseph Feuerstein, Silvana Bonilla, Sarah Flier, Javier Villafuerte-Gálvez

**Affiliations:** Beth Israel Deaconess Medical Center, Boston, Massachusetts; Beth Israel Deaconess Medical Center, Boston, Massachusetts; Beth Israel Deaconess Medical Center, Boston, Massachusetts; Boston Children's Hospital, Boston, Massachusetts; Beth Israel Deaconess Medical Center, Boston, Massachusetts; Beth Israel Deaconess Medical Center, Boston, Massachusetts

## Abstract

**Background:**

The BIDMC Optimization & Standardization for Treatment Outcomes in New England for *Helicobacter Pylori* (BOSTON-HP) study included 1002 patients with confirmed *Helicobacter pylori* (HP) via gastric histopathology from 2016-2022 to evaluate the effects of a Multidisciplinary Division Wide Program. Our previous data showed that demographic factors, such as race/ethnicity and preferred language, are associated with treatment, test-of-cure, and eradication outcomes. We hypothesize that given an unlikely even geographic distribution of demographic factors, HP outcomes may cluster geographically.

**Methods:**

The BOSTON-HP cohort patient addresses were transformed to georeferenced coordinates which were mapped using QGIS (3.30 's-Hertogenbosch) onto OpenStreetMap. We calculated the maximum Euclidean distance matrix to assign heatmap weights for each outcome, and calculated global spatial autocorrelation to evaluate the extent of spatial clustering.

**Results:**

Eight hundred and sixty-eight patients (86.6%) received treatment with confirmed eradication in 416 (47.9%) of them. A statistically significant positive spatial autocorrelation of clusters (Moran’s I = 0.080, p =0.003, z=2.787) was observed for the likelihood of treatment. Layering transportation and healthcare units revealed key clustering patterns (see Figures 1. & 2.). Clusters in the vicinity of Longwood Medical Area (LMA), and South Cove Medical Center (SCMC) were more likely to receive treatment, while those in the Brighton collegiate area (BCA) and along the Red Line Axis in Roxbury-Dorchester (RLRD) were less likely to receive treatment. Test-of-cure and eradication success were not statistically significant for clustering (Moran’s I = 0.007, p =0.388, z=0.284) (Moran’s I = 0.006, p =0.441, z=0.149), respectively.

**Figure:**
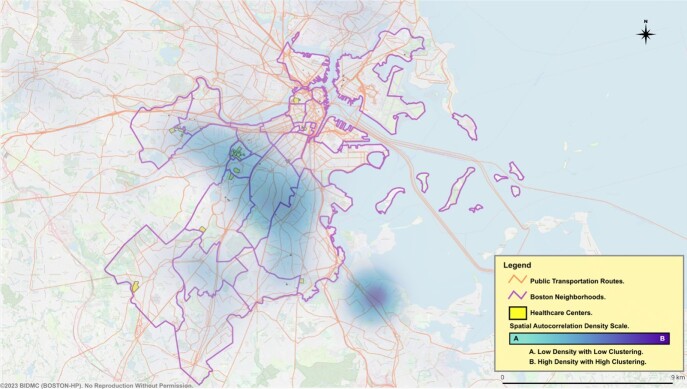
Figure 1. BOSTON-HP Treatment Cohort.

**Figure:**
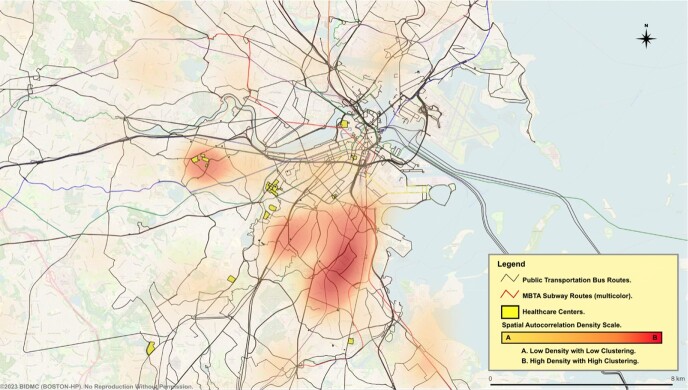
Figure 2. Untreated in the BOSTON-HP Cohort.

**Conclusion:**

A moderate and significant spatial correlation existed between the likelihood of HP treatment and the geographic location of the patient’s residence. Identification of groups for targeted quality improvement interventions (RLRD) and models of success within the system (SCMC) can help improve treatment outcomes and reduce geographic disparities.

**Disclosures:**

**Javier Villafuerte-Gálvez, MD**, Milky Way Biosciences: Partial Research Support.

